# Placenta Prævia, a Case Complicated with Twins

**Published:** 1888-10

**Authors:** Sam Cunningham

**Affiliations:** Elgin, Texas


### PLACENTA PRÆVIA.—A CASE.

By Sam Cunningham, M. D., Elgin, Texas.

[Read at Austin District Medical Society in June, '88.]

ON the morning of May 22, 1877, I was requested to see Mrs. L—— primipara; physique robust, who was taken in labor on the evening before at about 9 o'clock. I was told that the pains had only been light during the night, and did not increase in force and frequency until near daylight, when at about 7 o'clock, they increased to such a degree that the amniotic sack was ruptured, and I was hurriedly summoned, messenger also stating that she was flooding. Upon my arrival, I found her condition a most alarming one. She had certainly lost an immense amount of blood, as the bed under her was saturated, and she was pale, completely relaxed, with pulse very thready and weak. Upon digital examination, the funis was found prolapsed from the vaginal outlet several inches, and the right hand of the child was just emerging from the os uteri. Still, on passing the index and middle fingers into the os, which was nearly dilated, the placental mass was felt in close proximity with the arm. It seemed to be almost, if not entirely, detached, as was verified by its position, and by being able to sweep the fingers between it and the uterine wall. Hemorrhage still ensuing, the gravity of the case suggested immediate and radical interference; it was not deemed wise to subject the patient to further delay, lest the recurring hemorrhage might prove immediately fatal in her exhausted condition. As the orifice of the os was not entirely occluded by the presence of the arm and placenta, the vagina was tamponed with a tuft of absorbent cotton saturated in vinegar, and allowed to remain for a while, during which interval two drachm doses of ergot fld. ext. (Squibbs), was given 15 minutes apart, and also some brandy. Fearing the shock that might be produced by the introduction of the hand, chloroform was administered by inhalation to complete anæsthesia, notwithstanding the great loss of blood. After placing her across the bed, with hips resting on the side edge, the feet were fixed on two chairs and her legs supported by assistants. Turning and delivery being now in order, the tampon was removed; a large coagulum of blood followed its removal. All this time there was no pulsation in the cord. Pushing the arm back, the right hand was slowly and carefully introduced along the front of the child, until I could seize the feet, *whereupon was determined the existence of twins*, the head of the second child being close up to the feet that I had just grasped. Bringing the feet slowly down, version was easily performed, and delivery of first child effected with comparative ease, with exception of the shoulders and head. The arms being extended, were made to sweep over the face of the child.

Traction moderate was then made, but to no effect. The face was then bathed with cold water, with the view that the pain would return by overcoming the anæsthesia. Fortunately the pain was soon excited and by making traction with two fingers introduced into the mouth, together with extension of, or pressure on the occiput, I delivered her of a dead female child ; immediately after which followed the expulsion of the placenta attached. The umbilical cord was only about two feet in length. No hemorrhage ensuing at this time, I was inclined to think that there was more virtue in ergot than I had ever experienced before. Strange to say, at this stage, after so much exhaustion, the labor should put on so near a natural appearance. She lay quiet and apparently easy. On passing two fingers again into the os, the sensation imparted to the touch was that described by Playfair, "a little balloon," which was a separate and distinct bag of waters, pressing upon which could be felt the hard resistent head of the second child presenting. A drachm dose of ergot was again given both for its oxytocic as well as hemostatic effect. Permit me to say just here, however, that I am not in the habit of administering ergot as an oxytocic, as I believe much harm is often done thus used, for instance, hourglass contraction is often produced by an indiscreet use of the drug. Rupturing the bag of waters now presenting, the head was soon expelled, then a moment's rest, and a second pain propelled the body of the child. Respiration was established, as was immediately demonstrated by the crying of the child, which was a boy weighing seven pounds. The cord, which was about normal length, was at once ligated, and the child severed from the mother and handed to the nurse. On again turning to my patient, to my great sorrow I found the hemorrhage most alarming. More fear was felt of fainting and exhaustion than ever; the pulse was a mere thread, so weak and frequent that I could not count it. Three doses of ergot were given at short intervals, kneading being practiced through the abdominal parieties all the time, with the view of exciting uterine contraction. Traction was made on the cord, but to no avail. Some brandy and ergot were again given and the right hand was introduced, the vigina cleared of all clots which filled it, and the placenta scooped out of the thoroughly relaxed uterus with the fingers. The fingers were still kept in the uterus, which, by means of the pressure excited thus within the organ, and by the left hand over the abdomen, was under pretty thorough control. Small pieces of ice were at this time pushed into the vagina, and thence into the uterus.

The internal hand was not removed for some half hour or so, or until such an amount of contraction was felt as to remove all fear of further hemorrhage, at least for the time. The result was satisfactory; there was no more hemorrhage. To overcome the shock of so tedious an ordeal, a hypodermic of ⅓ grain morph. sulph., 1-30 gr. atrop sulph., and 15 gtt tinct. dig. was injected under the skin of the arm. She soon dropped off into a quiet slumber, and the pulse became better. Brandy and digitalis were given continually at short intervals, the action of which was quite perceptible on the circulation. After a reasonable length of time she was sponged off, and rid of all soiled garments, and put comfortably to bed, when she again resumed her slumbers and rested quietly during most of the day.

Being near by I saw her several times during the day. She would call for water occasionally. At 7 o'clock p. m. the pulse was 112 per minute, temperature 99 deg. F. Some clots of blood had escaped per vaginum, but no hemorrhage had occurred since the cessation that morning at 8 o'clock. Repeating the opiate above described, I left her, promising to see her next morning. On the morning of 23d arriving, was told that she had passed the night comfortably until near daylight, when she became restless and was unable to void the urine. A catheter was at once introduced and a pint of high-colored, strong amniotic urine escaped. The temperature at this time was 110 deg., pulse 116. There was a good deal of tenderness over the abdomen. Hot turpentine stupes were applied and the bowel opened by a dose of castor oil and turpentine.

Fearing that the fever would be protracted, and feeling the importance of regarding thorough antiseptic precautions, the following plan was adopted: Quin. Sulph. was given every few hours in five grain doses, until its effect was obtained; then the interval of giving lengthened so as to only keep up the stimulating effect of the antiperiodic. A metalic male catheter was introduced to the fundus, guided into the os by two fingers, introduced in the vagina, and being attached to a Davidson syringe, the parturient canal was thoroughly washed out with warm carbolized water. On returning in the evening, was told that she had been very thirsty, calling for water often during the day, but could not be induced to take any nourishment, such as broths, soft eggs and the like, that I had ordered given her. Temperature, 103½; pulse, 120. Skin rather cold, face pale and dejected. Some nourishment was forced upon her, and instructed to be given thus at regular intervals. The douche was again employed thoroughly, and as hot as could be borne, to the result of which I attributed the decline in the temperature to 102 deg. in an hour. The quinine was continued as at first, and a dose of brom. pot. and morphine was given to quiet nervousness,

Next morning, the 24th, on seeing her, I was gratified to find that she had passed a good night, and that the pulse, though 108 per minute, had a better volume; the temperature 100°. She stated that she felt better, but complained of pains over the abdomen, and an inability to void the urine freely. The warm douche was again used, and she passed the urine at the same time with ease, much tending to her comfort. The bowel was opened by an enema. Hot applications being continued during the day, eased the soreness, and stimulated the lochia, which had been scant for the last twenty-four hours. At 7 o'clock p. m., temperature 101° Fr.; pulse 112. No change in the above named treatment, only being sponged well with warm water, and linen changed. A dose of bro. pot. and morphine was left to be given in case of restlessness, which, however, was not given, as she slept well after being thus changed and made more comfortable in bed. The morning of the 25th found temperature 98½°, pulse 90. Quinine was ordered given thrice that day, as she could bear it exceedingly well; the douche continued, trusting it to the hand of a female attendant. Secretion of milk abundant; the child nursing and doing well. Seven o'clock p. m., temperature normal, pulse 84. From this time there was no more fever, and under the free use of iron, and other tonics, with an abundance of nutritious food, she made a more rapid convalescence than might have been expected.

To enumerate a little, I would beg to call attention to some of the facts mentioned in the case, in the history of which there are several features relative to its pathology and treatment worthy of consideration.

"Placenta prævia" in a primipara, which is rarely met with, as Playfair teaches that the multiparæ are most often the subjects of this serious complication. A case of twins. The difference in length of the two umbilical cords. The alarming hemorrhage, producing such complete exhaustion and relaxation. The kindly use of chloroform in this state of affairs, and such imperative indications for speedy delivery.

The most notable points in the history of treatment, however, were the prompt and remarkable action of ergot and the tampon in controlling hemorrhage for the time being, while chloroform was being administered and delivery affected. Last, but not least in importance, was the favorable influence of morph. and atrop. in allaying the severe shock existing, and finally the antiperiodic, tonic, and antiseptic powers of quinine, iron, and the carbolized douches, in restoring and sustaining the flagging powers of life by bringing about reaction, and so rapid a subsidence of the septicæmic symptoms.

---

FOR RINGWORM.—The *Chicago Medical Times* says a saturated solution of salicylic acid in collodion is a prompt cure for ringworm. The solution is painted on the affected parts once a day. One application generally suffices.

[We have found chrysophanic acid in collodion (elastic) very effective in ringworm.—ED.]

